# Sedentary behaviour and physical activity in bronchiectasis: a cross-sectional study

**DOI:** 10.1186/s12890-015-0046-7

**Published:** 2015-05-13

**Authors:** Judy M Bradley, Jason J Wilson, Kate Hayes, Lisa Kent, Suzanne McDonough, Mark A Tully, Ian Bradbury, Alison Kirk, Denise Cosgrove, Rory Convery, Martin Kelly, Joseph Stuart Elborn, Brenda O’Neill

**Affiliations:** Centre for Health and Rehabilitation Technologies, Institute for Nursing and Health Research, Ulster University, Newtownabbey, Northern Ireland UK; Northern Ireland Clinical Research Network: Respiratory Health, Belfast Health and Social Care Trust, Belfast, Northern Ireland UK; UKCRC Centre of Excellence for Public Health (Northern Ireland), Belfast, Northern Ireland UK; Centre for Public Health, School of Medicine, Dentistry and Biomedical Sciences, Queen’s University, Belfast, Northern Ireland UK; School of Psychological Sciences and Health, University of Strathclyde, Glasgow, Scotland UK; Southern Health and Social Care Trust, Craigavon Area Hospital, Craigavon, Northern Ireland UK; Western Health and Social Care Trust, Altnagelvin Area Hospital, Derry, Northern Ireland UK; Centre for Infection and Immunity, School of Medicine, Dentistry and Biomedical Sciences, Queen’s University, Belfast, Northern Ireland UK

**Keywords:** Bronchiectasis, Physical activity, Sedentary behaviour

## Abstract

**Background:**

The impact of bronchiectasis on sedentary behaviour and physical activity is unknown. It is important to explore this to identify the need for physical activity interventions and how to tailor interventions to this patient population. We aimed to explore the patterns and correlates of sedentary behaviour and physical activity in bronchiectasis.

**Methods:**

Physical activity was assessed in 63 patients with bronchiectasis using an ActiGraph GT3X+ accelerometer over seven days. Patients completed: questionnaires on health-related quality-of-life and attitudes to physical activity (questions based on an adaption of the transtheoretical model (TTM) of behaviour change); spirometry; and the modified shuttle test (MST). Multiple linear regression analysis using forward selection based on likelihood ratio statistics explored the correlates of sedentary behaviour and physical activity dimensions. Between-group analysis using independent sample t-tests were used to explore differences for selected variables.

**Results:**

Fifty-five patients had complete datasets. Average daily time, mean(standard deviation) spent in sedentary behaviour was 634(77)mins, light-lifestyle physical activity was 207(63)mins and moderate-vigorous physical activity (MVPA) was 25(20)mins. Only 11% of patients met recommended guidelines. Forced expiratory volume in one-second percentage predicted (FEV_1_% predicted) and disease severity were not correlates of sedentary behaviour or physical activity. For sedentary behaviour, decisional balance ‘pros’ score was the only correlate. Performance on the MST was the strongest correlate of physical activity. In addition to the MST, there were other important correlate variables for MVPA accumulated in ≥10-minute bouts (QOL-B Social Functioning) and for activity energy expenditure (Body Mass Index and QOL-B Respiratory Symptoms).

**Conclusions:**

Patients with bronchiectasis demonstrated a largely inactive lifestyle and few met the recommended physical activity guidelines. Exercise capacity was the strongest correlate of physical activity, and dimensions of the QOL-B were also important. FEV_1_% predicted and disease severity were not correlates of sedentary behaviour or physical activity. The inclusion of a range of physical activity dimensions could facilitate in-depth exploration of patterns of physical activity. This study demonstrates the need for interventions targeted at reducing sedentary behaviour and increasing physical activity, and provides information to tailor interventions to the bronchiectasis population.

**Trial registration:**

NCT01569009 (“Physical Activity in Bronchiectasis”)

**Electronic supplementary material:**

The online version of this article (doi:10.1186/s12890-015-0046-7) contains supplementary material, which is available to authorized users.

## Background

There is strong evidence that adherence to physical activity guidelines is associated with health benefits and reduced mortality in both healthy and chronic disease populations [1,2]. There is no specific evidence that physical activity is beneficial in bronchiectasis; however it is strongly related to mortality and lung health in other respiratory conditions such as chronic obstructive pulmonary disease (COPD) and cystic fibrosis [3-5]. Promoting physical activity has been proposed as a key component of care in chronic respiratory disease [2,3,6,7]. International recommendations for the whole population promote a minimum of 150 minutes of at least moderate physical activity per week (accumulated in at least 10-minute bouts) and a restriction on extended periods of sedentary behaviour for promoting and maintaining health [1]. The impact of bronchiectasis on sedentary behaviour and physical activity is unknown. It is important to explore this to identify the need for physical activity interventions and how to tailor interventions to this patient population.

Objective assessment of sedentary behaviour and physical activity using activity monitors has been recommended in preference to questionnaires [7-9]. In this study, we chose to use the ActiGraph activity monitor as it is one of the most studied activity monitors with demonstrated reliability and validity in respiratory disease populations [10,11]. The ActiGraph activity monitor measures many different physical activity dimensions but currently there is limited research to inform clinicians on which of these variables are most useful. Van Remoortel and colleagues have proposed that time spent in different physical activity intensities, energy expenditure and step counts should all be considered to provide a comprehensive assessment [12]. The ActiGraph activity monitor also measures time spent in sedentary behaviours such as lying and sitting. Previous research has highlighted how sedentary behaviour has an important role on patients’ clinical progression [13].

A range of clinical characteristics (disease severity, exercise capacity, health-related quality-of-life (HRQoL) and symptoms) have been shown to impact on sedentary behaviour and physical activity in other respiratory conditions [13-16]. However, their impact in bronchiectasis is unknown.

Additionally psychological and behavioural factors may also have an impact on sedentary behaviour and physical activity. An adaption of the transtheoretical model (TTM) of behaviour change can be used as a framework to identify why patients with bronchiectasis engage in physical activity or not, and when and how individuals are likely to change their physical activity behaviour [17,18]. The TTM constructs include the stages of change, self-efficacy, decisional balance and both cognitive and behavioural processes of change (more details included in Table 1 and Additional file 1). The TTM assumes that behaviour change is a dynamic process rather than an all-or-nothing phenomenon [19]. However, specific data in patients with bronchiectasis using the TTM is not yet available [18]. Understanding the links between physical activity and sedentary behaviour, and clinical and psychological characteristics will potentially inform the development of future physical activity interventions.

**Table 1:** **Description of each component of the transtheoretical model (TTM)**

**TTM construct** ** [** 18 **]**	**Description** **[** 18 **]**
**Stage of change**	
Pre-contemplation	No intention to engage in regular physical activity
Contemplation	Intend to engage in regular physical activity in next 6 months
Preparation	Immediate intentions and commitment to engage in regular physical activity
Action	Initiated engagement in regular physical activity in last 6 months
Maintenance	Maintained engagement of regular physical activity for longer than 6 months
**Self-efficacy**	Personal confidence towards physical activity commitment when: Tired/In a bad mood/Do not have time/On vacation/It is raining or snowing/Having respiratory symptoms*
**Decisional balance**	
Pros	Perceived benefits of engaging in regular physical activity
Cons	Perceived barriers to engaging in regular physical activity
**Cognitive processes of change**	
Increasing knowledge	Finding information on the benefits of physical activity and the current recommendations for physical activity
Being aware of risk	Concern for the risks of being physically inactive
Caring about consequences	Realising social and environmental benefits that physical activity has
Comprehending benefits	Assessing physical activity status and the values related to physical activity
Increasing healthy opportunities	Awareness, availability and acceptance by the individual of physical activity in the society
**Behavioural processes of change**	
Substituting alternatives	Substituting inactive options for active options
Enlisting social support	Seeking out social support to increase and maintain physical activity
Rewarding oneself	Providing rewards for being more active
Committing oneself	Setting goals and making commitments for physical activity
Reminding oneself	Controlling factors that have a negative effect on physical activity to prevent relapse and using stimuli to increase physical activity level

*Question on ‘having respiratory symptoms’ was added to the original five questions.

The overall aim of this research was to explore sedentary behaviour and physical activity and correlates of these behaviours in patients with bronchiectasis. Specific objectives of this research were to explore patterns of physical activity in patients with bronchiectasis and determine if patients meet the current physical activity guidelines; and to examine the relationship between physical activity levels of patients with bronchiectasis and clinical characteristics (disease severity, exercise capacity, HRQoL and other symptoms of their disease) and constructs of the TTM (stages of change, self-efficacy, decisional balance and processes of change).

The research hypothesis was that patients with bronchiectasis would have high levels of sedentary behaviour and low levels of physical activity and these would be related to clinical characteristics and constructs of the TTM. More specifically, it was hypothesised that lower sedentary behaviour and higher levels of physical activity would be related to greater exercise capacity, greater lung function, better HRQoL and higher self-efficacy, perceiving more benefits of physical activity and using more processes of change.

## Methods

### Participant selection

Due to the exploratory nature of the study, a sample of 63 patients was feasible based on constraints of time (one-year time period) and availability of patients. Consecutive patients attending respiratory clinics at the three selected hospital sites were screened for eligibility. Inclusion criteria were: aged ≥18 years, diagnosis of bronchiectasis confirmed by high-resolution CT/CT, ≤10 pack-year smoking history, clinically stable (no pulmonary exacerbation [more details in Additional file 1] and no significant change in symptoms or medication in the last four weeks) and sputum bacteriology completed over the past three months. Exclusion criteria were: current severe haemoptysis, pregnancy or any other concomitant condition that would prevent participation. Study recruitment occurred over 12 months and patients were recruited across all seasons. The study was approved by Northern Ireland Research Ethics Committees (Ethics Approval Reference: 12/NI/0044) and research departments of all participating hospitals. Written informed consent was obtained from all study patients.

### Study design

**Figure 1:**
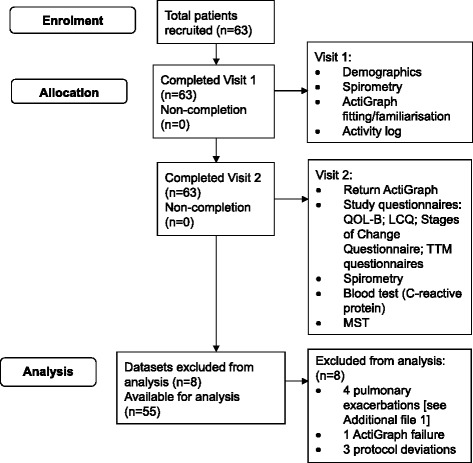
Study flow diagram showing patient enrolment, allocation and analysis. Abbreviations: QOL-B - Quality of Life Questionnaire-Bronchiectasis; LCQ - Leicester Cough Questionnaire; Transtheoretical model (TTM) questionnaires - Marcus’s Self-Efficacy Questionnaire, Marcus’s Decisional Balance Questionnaire, Marcus’s Processes of Change Questionnaire; MST - Modified Shuttle Test.

This was a cross-sectional study using quantitative methodology (Figure 1). Patients attended Visit 1 where age and gender were recorded and an assessment of body mass index (BMI) and spirometry was conducted. The ActiGraph was attached and worn for seven consecutive days following Visit 1. Eight days later, patients attended Visit 2 where they returned the ActiGraph and activity log and completed study questionnaires, spirometry, a blood test for C-reactive protein, and the Modified Shuttle Test (MST) [20].

### Clinical measurements

Height and weight were measured in light clothing and without shoes using SECA digital scales and stadiometer. Spirometry was assessed using MicroLab spirometer ML3500 and classified according to American Thoracic Society/European Respiratory Society Guidelines [21]. Disease severity was calculated using the Bronchiectasis Severity Index (BSI) to identify patients at risk of exacerbations, hospitalisations and mortality [22] (see Additional file 1).

Physical activity was measured using the ActiGraph GT3X+ accelerometer (ActiGraph, Pensacola, Florida). Patients wore the ActiGraph during all waking hours for seven consecutive days following Visit 1. The ActiGraph was worn on an elastic belt and patients were instructed to position the ActiGraph on the anterior axillary line of the hip on their dominant side. They were advised to remove the ActiGraph before sleeping and prior to water-based activities. Patients recorded any non-wear periods in a daily activity log. The ActiGraph was initialised using the manufacturer’s software (ActiLife version 6.8.0) to record movement in counts per minute summed over 15-second epochs. On Visit 1, patients were offered daily, alternate-day or once-weekly reminders to wear the ActiGraph. Each patient’s ActiGraph data was considered valid if there were ≥10 hours of wear-time per day for ≥5 days, to include a Saturday or Sunday [23,24]. Using ActiLife software, wear-time validation was applied using established parameters which allowed for a 2-minute interval of non-zero counts with an up/downstream 30 minutes of consecutive zero counts window [25]. Patient-completed activity logs were cross-checked to explore non-wear periods. Details of sedentary behaviour and physical activity dimensions are included in Additional file 1.

Study questionnaires were administered and completed during Visit 2. The questionnaires included: Quality of Life Questionnaire-Bronchiectasis (QOL-B) [26], Leicester Cough Questionnaire (LCQ) [27], Stages of Change Questionnaire [28], Marcus’s Self-Efficacy Questionnaire (with additional disease-specific question) [29], Marcus’s Decisional Balance Questionnaire [30] and Marcus’s Processes of Change Questionnaire [31]. Questionnaires were completed in a standardised order and were cross-checked by researchers to ensure no missing data (see Additional file 1).

Exercise capacity was measured using the MST, a progressive 15-stage exercise field test which is based on a standardised protocol [20]. The MST was performed twice with ≥20 minute rest between tests. The greatest distance completed in either MST was used for analysis. The MST has been shown to have good reliability and validity [32].

### Statistical analysis

Descriptive statistics were used to summarise demographic and clinical characteristics and physical activity intensity categories.

Multiple linear regression analysis using forward selection based on likelihood ratio statistics was completed with sedentary behaviour and physical activity dimensions as the dependent variables. Dependent variables included daily sedentary behaviour time and different physical activity dimensions (see Additional file 1). Independent variables entered into the model included: BSI score, age, gender, BMI, FEV_1_% predicted, MST, LCQ domains, QOL-B domains except QOL-B Treatment Burden (this response is not scored for every patient) and constructs of the TTM (Marcus’s Self-Efficacy average score, Marcus’s Decisional Balance ‘pros’ and ‘cons’ scores, Marcus’s Processes of Change cognitive and behavioural average scores). As this was an exploratory study, no correction was made for multiplicity. The significance levels are therefore descriptive rather than inferential.

Between-group analysis using independent sample t-tests were used to explore differences for selected variables. All statistical analyses were performed using SPSS version 20.0.0 (IBM). Unless otherwise stated, summary data are reported as mean(SD) and statistical significance as p < 0.05.

## Results

Sixty-three patients completed the study visits. Eight datasets were not valid, leaving fifty-five datasets for analysis (Figure 1 and Table 2). BSI scores categorised patients’ disease severity as mild (49%), moderate (33%) and severe (18%) [9]. In general, bronchiectasis impacted on patients’ HRQoL across most QOL-B domains. The QOL-B indicated that patients perceived a high Treatment Burden and a low Health Perception. They had good Emotional Functioning and were not largely affected by Respiratory Symptoms. The LCQ indicated that chronic cough impacted on HRQoL, with highest perceived impact on the Physical domain (lowest LCQ domain score). C-reactive protein at study entry was 4(4)mg/L (Table 2).

**Table 2:** **Demographic and clinical characteristics of patients with bronchiectasis (n = 55)**

Age (years)	63 (10)
Gender (male / female)	22 [40]/33 [60]
BMI (kg/m^2^)	27 (4)
FEV_1_ (litres)	2 (1)
FEV_1_ (% predicted)	76 (21)
FVC (litres)	3 (1)
FVC (% predicted)	94 (19)
FEF%	38 (22)
FEF_25–75_ (litres)	1 (0.8)
C-Reactive Protein (mg/L)	4 (4)
Disease severity (%)*	
Mild	27 [49]
Moderate	18 [33]
Severe	10 [18]
**Smoking history**	
Never (%)	46 [84]
Ex-smoker (%)	9 [16]
**Antibiotic courses **	
Number of oral antibiotic courses within last year	3 (2)
Number of IV antibiotic courses within last year	0-3 (range)
**QOL-B (0–100, 0 worst to 100 best)**	
Physical Functioning	59 (31)
Role Functioning	56 (12)
Vitality	63 (13)
Emotional Functioning	83 (17)
Social Functioning	60 (23)
Treatment Burden (n = 41)	39 (13)
Health Perception	45 (16)
Respiratory Symptoms	70 (19)
**LCQ (1–7, 1 worst to 7 best)**	
Physical	4.96 (1.43)
Psychological	5.27 (1.52)
Social	5.50 (1.29)
LCQ total score (range from 3 to 21)	15.72 (3.99)

Results are Mean (SD) or Frequency [%].

*Abbreviations:* *BMI* Body Mass Index, *FEF* Forced Expiratory Flow, *FEF*_*25–75*_ Forced Expiratory Flow between 25% to 75%, *FEV*_*1*_ *%* predicted Forced Expiratory Volume in one-second percentage predicted, *FVC* Forced Vital Capacity (% predicted), *LCQ* Leicester Cough Questionnaire, *QOL-B* Quality of Life in Bronchiectasis.

*Disease severity based on Bronchiectasis Severity Index [22].

### Sedentary behaviour and physical activity levels

Average daily time spent in sedentary behaviour was 634(77)mins, light-lifestyle physical activity was 207(63)mins and moderate-vigorous physical activity (MVPA) was 25(20)mins. Only 11% of patients met the recommended physical activity guidelines of ≥150mins of at least moderate physical activity per week [1]. Patients completed 6001(2780) daily steps and 232(75)mins of daily total physical activity. Using the graduated step-based physical activity index, 42% of patients were classified as inactive, 29% as low active and 29% as somewhat active and above [33]. Mean distance covered in the MST was 511(273)metres (Table 3).

**Table 3:** **Sedentary behaviour (ActiGraph), physical activity (ActiGraph) and exercise capacity (MST) for patients with bronchiectasis (n = 55)**

**Average times in sedentary behaviour and different physical activity intensities:**	
Sedentary behaviour time (mins/day)	634 (77)
Light-lifestyle physical activity time (mins/day)	207 (63)
Total MVPA time (mins/day)	25 (20)
MVPA_10+_ time (mins/week)	44 (64)
MVPA_10+_ time (mins/day)	6 (9)
Activity energy expenditure (kcals/day)	309 (183)
Daily step counts	6001 (2780)
Total physical activity (mins/day)	232 (75)
Physical activity category Inactive [%]	23 [42]
Physical activity category Low active [%]	16 [29]
Physical activity category Somewhat active and above [%]	16 [29]
**Exercise capacity:**	
MST (metres)	511 (273)

Results are Mean (SD) or Frequency [%].

ActiGraph physical activity categories: Inactive (<5000 steps per day), low active (5000–7499 steps per day) and somewhat active and above (≥7500 steps per day).

*Abbreviations:* *kcals/day* kilocalories per day, *MVPA* moderate-vigorous physical activity, *MVPA*_*10+*_ MVPA accumulated in ≥10-minute bouts, *mins/day* minutes per day, *mins/week* minutes per week, *MST* Modified Shuttle Test.

### Correlates of sedentary behaviour and physical activity

Table 4 shows variables for inclusion in the regression analysis with a p-value below 5%. The correlates selected in this study explained 10-38% of the variance in sedentary behaviour and physical activity. Forced expiratory volume in one-second percentage predicted (FEV_1_% predicted) and disease severity (BSI score) were not correlates of sedentary behaviour or any physical activity variable. The MST was not a correlate of sedentary behaviour time. For sedentary behaviour time, decisional balance ‘pros’ score was a correlate variable; with those who were more sedentary observing less benefits of physical activity. For physical activity variables, the MST was the most strongly related correlate variable. For MVPA accumulated in ≥10-minute bouts, QOL-B Social Functioning was also a correlate variable; with those who completed more MVPA in ≥10-minute bouts having higher Social Functioning. For activity energy expenditure, BMI and QOL-B Respiratory Symptoms were also correlate variables; with those who had greater activity energy expenditure having a higher BMI and worse Respiratory Symptoms (Table 4).

**Table 4:** **Correlate variables for sedentary behaviour and physical activity for patients with bronchiectasis (n = 55)**

**Dependent variable**	**Correlate variable**	**Unstandardised coefficients B(SE)**	**R** ^**2**^ **adjusted**	**p value**
Daily sedentary behaviour time	Marcus’s Decisional Balance ‘pros’ score	−28.964 (10.609)	0.107	0.009
Daily light-lifestyle PA time	No correlates	---	---	---
Daily total MVPA time	MST	0.037 (0.008)	0.258	0.001
Daily MVPA_10+_ time	QOL-B Social Functioning	0.162 (0.050)	0.149	0.002
	MST	0.009 (0.004)	0.207	0.032
Daily AEE	MST	0.351 (0.077)	0.269	0.001
	BMI	12.769 (4.767)	0.345	0.010
	QOL-B Respiratory Symptoms	−2.215 (1.074)	0.384	0.044
Daily step counts	MST	5.813 (1.127)	0.322	0.001
Daily total PA time	MST	0.088 (0.035)	0.087	0.016

*Abbreviations:* *AEE* activity energy expenditure, *BMI* Body Mass Index, *MST* Modified Shuttle Test, *MVPA* moderate-vigorous physical activity, *MVPA*_*10+*_ MVPA accumulated in ≥10-minute bouts, *PA* physical activity, *QOL-B* Quality of Life-Bronchiectasis.

Patients with moderate/severe disease (BSI score: ≥5) spent significantly less time in daily total MVPA time, had lower activity energy expenditure, fewer daily step counts and achieved lower MST distance than those with mild disease (BSI score: ≤4) (Table 5).

**Table 5:** **Differences across disease severity for sedentary behavior, physical activity and exercise capacity for patients with bronchiectasis**

	**Disease severity: Mild BSI score ≤ 4(n = 27)**	**Disease severity: Moderate/severe BSI score ≥ 5(n = 28)**
Sedentary behavior time (mins/day)	632 (64)	635 (88)
Light-lifestyle physical activity time (mins/day)	210 (55)	204 (71)
Total MVPA time (mins/day)	32 (19)	18 (18)^a^
MVPA_10+_ time (mins/day)	8 (10)	5 (8)
Activity energy expenditure (kcals/day)	390 (173)	231 (159)^b^
Daily step counts	6898 (2783)	5137 (2532)^c^
Total physical activity time (mins/day)	242 (65)	221 (84)
MST (metres)	593 (323)	432 (199)^d^

Results are Mean (SD).

Note: Disease severity expressed as Bronchiectasis Severity Index score [22].

*Abbreviations:* *kcals/day* kilocalories per day, *MVPA* moderate-vigorous physical activity, *MVPA*_*10+*_ MVPA accumulated in bouts ≥10 minutes, *mins/day* minutes per day, *MST* Modified Shuttle Test.

^a^Daily Total MVPA: significant difference between groups (p = 0.005).

^b^Daily Activity Energy Expenditure: significant difference between groups (p = 0.001).

^c^Daily Step Counts: significant difference between groups (p = 0.017).

^d^MST: significant difference between groups (p = 0.030).

Fifty-five percent of patients reported that they were in an ‘inactive’ stage of change (pre-contemplation, contemplation or preparation stages) while 45% reported themselves to be in an ‘active’ stage of change (action or maintenance stages) in relation to their participation in physical activity (Table 6). Patients reported reduced confidence when faced with situations that could impact on their ability to participate in physical activity; being most confident that they could be physically active when on holiday and least confident when they had respiratory symptoms. They also perceived both benefits (‘pros’) and barriers (‘cons’) to physical activity. Decisional balance scores (perceived benefits minus perceived barriers) showed patients perceived marginally more benefits. Overall, patients used cognitive and behavioural strategies equally in their physical activity behaviour (Table 6).

**Table 6:** **Stages of change scores and TTM questionnaire scores for patients with bronchiectasis**

**Stage of change:**	
Stage 1 pre-contemplation [%]	4 [7]
Stage 2 contemplation [%]	6 [11]
Stage 3 preparation [%]	20 [36]
Stage 4 action [%]	3 [6]
Stage 5 maintenance [%]	22 [40]
**Marcus’s self-efficacy:**	
**(1–5, 1 not at all confident to 5 very confident in being active)**	
When tired	2.27 (0.95)
When in a bad mood	2.96 (1.19)
When do not have time	2.53 (1.07)
When on vacation	3.35 (1.22)
When raining/snowing	2.33 (1.25)
When having respiratory symptoms	1.65 (0.97)
Mean of all 6 self-efficacy domains	2.52 (0.48)
**Marcus’s decisional balance:**	
**(scores > 0 indicate perceptions of more benefits than barriers in being active, scores < 0 indicate perceptions of more barriers than benefits in being active)**	
Pros (1–5, higher scores perceive more benefits in being active)	3.53 (0.93)
Cons (1–5, higher scores perceive more barriers in being active)	2.62 (0.75)
Overall decisional balance score (difference between pros minus cons)	0.91 (1.01)
**Marcus’s processes of change:**	
**(1–5, higher scores indicate greater usage of strategies to become more active)**	
*Cognitive Processes*	
Increasing knowledge	2.49 (0.81)
Being aware of risks	2.35 (1.01)
Caring about consequences to others	2.52 (1.04)
Comprehending benefits	3.16 (1.01)
Increasing healthy opportunities	2.34 (0.94)
Cognitive processes mean	2.57 (0.78)
*Behavioural Processes*	
Substituting alternatives	2.99 (0.98)
Enlisting social support	2.40 (0.93)
Rewarding oneself	2.44 (0.94)
Committing oneself	3.07 (0.95)
Reminding oneself	1.92 (0.72)
Behavioural processes mean	2.56 (0.70)

Results are Mean (SD) or Frequency [%].

## Discussion

This is the first study to report patterns of sedentary behaviour and physical activity in bronchiectasis. The results demonstrate a more sedentary and less active profile for people with bronchiectasis compared to the recommended guidelines for physical activity. These findings are important as recent research has suggested a link with inactivity and decreased survival, poorer HRQoL and increased healthcare utilisation in chronic disease populations such as COPD and diabetes [3,4,33-36]. Furthermore, there is increasing evidence that a high level of sedentary behaviour is associated with adverse health outcomes in chronic disease populations [36-38].

To contextualise these study findings, we have compared our bronchiectasis data to similar ActiGraph data available for English, Swedish and USA healthy populations [39-43] and to another respiratory population [16] (see Additional file 2). Albeit the healthy data sets are more heterogeneous in terms of age and ethnicity, some important contrasts emerge. Patients with bronchiectasis appear to have similar levels of sedentary behaviour and physical activity compared to the English healthy population [42]; both populations fall well below recommended guidelines for physical activity [1]. Patients with bronchiectasis appear to be more sedentary and less physically active compared to healthy Swedish and USA populations [39-41,43]. USA population-based ActiGraph data is available in COPD. Patients with bronchiectasis appear to have a similar sedentary behaviour and physical activity profile; despite being younger in age [16]. When designing physical activity interventions in bronchiectasis, researchers may need to consider the impact of patients’ baseline sedentary behaviour and physical activity levels as well as current and new symptoms.

We hypothesised that lower levels of sedentary behaviour and higher levels of physical activity would be related to greater exercise capacity, greater lung function, better HRQoL and higher self-efficacy, perceiving more benefits of physical activity and using more processes of change. FEV_1_% predicted and BSI score did not correlate with sedentary behaviour time or physical activity variables highlighting that neither of these assessments should be used clinically as indicators of either sedentary behaviour or physical activity. Whilst MST did not predict sedentary behavior, MST consistently correlated with physical activity variables. This association between physical activity and exercise capacity has previously been demonstrated in bronchiectasis [44] and highlights the potential importance of exercise interventions, such as pulmonary rehabilitation, to improve physical activity levels in patients with bronchiectasis. Based on positive findings from five key pulmonary rehabilitation studies in bronchiectasis [45-49], recent British Thoracic Society Guidelines for Pulmonary Rehabilitation recommend referral to pulmonary rehabilitation for patients with bronchiectasis who have breathlessness affecting their activities of daily living [6]. The most recent of these studies by Lee et al. [49] recruited patients with a very similar demographic profile into a quality randomised controlled trial of eight weeks of pulmonary rehabilitation versus control and demonstrated that pulmonary rehabilitation was associated with short-term improvement in exercise capacity, dyspnoea and fatigue; although these improvements were not sustained at 12-month follow-up. The positive effects of pulmonary rehabilitation on exercise capacity across chronic respiratory conditions have been shown to consistently diminish over time [6]. With limited access to maintenance programmes, alternative strategies to reduce sedentary behaviour and/or increase and sustain physical activity may be important. Unfortunately, as with earlier studies, physical activity was not included as an outcome measure and further research is needed to establish whether changes in exercise capacity translate to changes in physical activity or whether physical activity needs to be specifically targeted in bronchiectasis.

There were important differences in the predictors of sedentary behaviour versus physical activity. In fact, decisional balance ‘pros’ score was the only correlate of sedentary behaviour suggesting that sedentary behaviour in bronchiectasis could be influenced more by psychological factors rather than physiological factors. The data shows that it is important to assess patients’ sedentary behaviour and physical activity levels directly. We also propose that it may be important to focus on behaviour change techniques and other behavioural strategies such as motivational interviewing [50] rather than exercise training alone if targeting a decrease in sedentary behaviour as well as improved physical activity levels in patients with bronchiectasis.

Although activity energy expenditure was estimated using equations developed for healthy populations, an interesting relationship emerged with QOL-B Respiratory Symptoms scores. Patients with higher activity energy expenditure appeared to have worse respiratory symptoms. Patients with chronic respiratory disease potentially have an increased oxygen cost of breathing compared with healthy populations due to respiratory dynamics [51].

In COPD, recent research suggests that higher physical activity levels are associated with higher self-efficacy and less depressive symptoms in patients with COPD [52]. We have shown that patients with bronchiectasis perceived a range of barriers to participation in physical activity, with those who were more sedentary perceiving more barriers. They employed a range of cognitive and behavioural strategies to modify their physical activity behaviour. The most employed strategies were: realising benefits of being physically active, making commitments to be physically active and replacing inactive choices with active choices. Future intervention studies could focus on optimising frequently used strategies as well as considering the value of less commonly used strategies to support patients in altering their physical activity behaviour.

A major strength of this study was the use of validated instruments to assess physical activity, exercise capacity and HRQoL in a bronchiectasis population. This facilitated rigorous exploration of the correlates of sedentary behaviour and physical activity in bronchiectasis. One limitation may be that due to the exploratory nature of this study, no sample size calculation was performed. Nevertheless, this exploration has provided a useful insight into understanding correlates of sedentary behaviour and physical activity in bronchiectasis.

## Conclusions

In summary, many patients with bronchiectasis demonstrated a largely inactive lifestyle and few met the recommended physical activity guidelines. FEV_1_% predicted and disease severity were not correlates of sedentary behaviour or physical activity. Exercise capacity was the strongest correlate of physical activity, and dimensions of the QOL-B were also important. Despite patients understanding the benefits of physical activity, many reported low levels of self-confidence in physical activity in certain situations, particularly when experiencing respiratory symptoms. This study highlights the need for physical activity interventions in bronchiectasis and provides information to tailor interventions to this patient population.

## Additional files

Additional file 1:
**Additional details of methods and materials used in the study.**
Additional file 2:
**Comparing sedentary behaviour and physical activity in patients with bronchiectasis to healthy and COPD populations.**

## Abbreviations

BMI: Body mass index
BSI: Bronchiectasis severity index
COPD: Chronic obstructive pulmonary disease
FEV_1_% predicted: Forced expiratory volume in one-second percentage predicted
HRQoL: Health-related quality of life
LCQ: Leicester cough questionnaire
MVPA: Moderate-vigorous physical activity
MST: Modified Shuttle Test
QOL-B: Quality of Life Questionnaire-Bronchiectasis
TTM: Transtheoretical model

## References

[1] O’Donovan G, Blazevich AJ, Boreham C, Cooper AR, Crank H, Ekelund U (2010). The ABC of physical activity for health: a consensus statement from the British Association of Sport and Exercise Sciences. J Sports Sci..

[2] Arne M, Janson C, Janson S, Boman G, Lindqvist U, Berne C (2009). Physical activity and quality of life in subjects with chronic disease: chronic obstructive pulmonary disease compared with rheumatoid arthritis and diabetes mellitus. Scand J Prim Health Care..

[3] Waschki B, Kirsten A, Holz O, Müller K-C, Meyer T, Watz H (2011). Physical activity is the strongest predictor of all-cause mortality in patients with COPD: a prospective cohort study. Chest..

[4] Garcia-Rio F, Rojo B, Casitas R, Lores V, Madero R, Romero D (2012). Prognostic value of the objective measurement of daily physical activity in patients with COPD. Chest..

[5] Schneiderman JE, Wilkes DL, Atenafu EG, Nguyen T, Wells GD, Alarie N (2014). Longitudinal relationship between physical activity and lung health in patients with cystic fibrosis. Eur Respir J..

[6] Bolton CE, Bevan-Smith EF, Blakley JD, Crowe P, Elkin SL, Garrod R (2013). British Thoracic Society guideline on pulmonary rehabilitation in adults. Thorax..

[7] Troosters T, van der Molen T, Polkey M, Rabinovich RA, Vogiatzis I, Weisman I (2013). Improving physical activity in COPD: towards a new paradigm. Respir Res..

[8] Shephard R (2003). Limits to the measurement of habitual physical activity by questionnaires. Br J Sports Med..

[9] Prince S, Adamo K, Hamel M, Hardt J, Gorber S, Tremblay M (2008). A comparison of direct versus self-report measures for assessing physical activity in adults: a systematic review. Int J Behav Nutr Phys Act..

[10] Rabinovich RA, Louvaris Z, Raste Y, Langer D, Van Remoortel H, Giavedoni S (2013). Validity of physical activity monitors during daily life in patients with COPD. Eur Respir J..

[11] Bradley JM, O’Neill B, Kent, L, Hulzebos EHJ, Arets B, Hebestreit H: Physical activity assessment in cystic fibrosis: a position statement endorsed by the European Cystic Fibrosis Society Board. In press. 10.1016/j.jcf.2015.05.011 26219990

[12] Van Remoortel H, Giavedoni S, Raste Y, Burtin C, Louvaris Z, Gimeno-Santos E (2012). Validity of activity monitors in health and chronic disease: a systematic review. Int J Behav Nutr Phys Act..

[13] Pitta F, Troosters T, Spruit MA, Probst VS, Decramer M, Gosselink R (2005). Characteristics of physical activities in daily life in chronic obstructive pulmonary disease. Am J Respir Crit Care Med..

[14] Troosters T, Langer D, Vrijsen B, Segers J, Wouters K, Janssens W (2009). Skeletal muscle weakness, exercise tolerance and physical activity in adults with cystic fibrosis. Eur Respir J..

[15] Troosters T, Sciurba F, Battaglia S, Langer D, Valluri SR, Martino L (2010). Physical inactivity in patients with COPD, a controlled multi-center pilot-study. Respir Med..

[16] Park SK, Richardson CR, Holleman RG, Larson JL (2013). Physical activity in people with COPD, using the National Health and Nutrition Evaluation Survey (NHANES) dataset (2003–2006). Heart Lung..

[17] Prochaska JO, DiClemente CC (1983). Stages and processes of self-change of smoking: toward an integrative model of change. J Consult Clin Psychol..

[18] Marcus BH, Forsyth LH (2009). Motivating people to be physically active.

[19] Marshall SJ, Biddle SJ (2001). The transtheoretical model of behavior change: a meta-analysis of applications to physical activity and exercise. Ann Behav Med..

[20] Bradley JM, Howard JL, Wallace ES, Elborn JS (1999). Validity of a modified shuttle test in adult cystic fibrosis. Thorax..

[21] Miller MR, Hankinson J, Brusasco V, Burgos F, Casaburi R, Coates A (2005). Standardisation of spirometry. Eur Respir J..

[22] Chalmers JD, Goeminne P, Aliberti S, McDonnell MJ, Lonni S, Davidson J (2014). The Bronchiectasis Severity Index: an international derivation and validation study. Am J Respir Crit Care Med..

[23] Gretebeck RJ, Montoye HJ (1992). Variability of some objective measures of physical activity. Med Sci Sports Exerc..

[24] Trost SG, McIver KL, Pate RR (2005). Conducting accelerometer-based activity assessments in field-based research. Med Sci Sports Exerc..

[25] Choi L, Liu Z, Matthews CE, Bouchowski MS (2011). Validation of accelerometer wear and non-wear time classification algorithm. Med Sci Sports Exerc..

[26] Quittner AL, O’Donnell AE, Salathe MA, Lewis SA, Xiaoming L, Montgomery AB (2015). Quality of life Questionnaire-Bronchiectasis: final psychometric analyses and determination of minimal important difference scores. Thorax..

[27] Birring SS, Prudon B, Carr AJ, Singh SJ, Morgan MDL, Pavord ID (2003). Development of a symptom specific health status measure for patients with chronic cough: the Leicester Cough Questionnaire (LCQ). Thorax..

[28] Loughlan C, Mutrie N (1995). Recruitment of sedentary NHS staff for a workplace exercise programme using an adapted “stages of change” exercise questionnaire. J Sports Sci..

[29] Marcus BH, Selby VC, Niaura RS, Rossi JS (1992). Self-efficacy and the stages of exercise behaviour change. Res Q Exerc Sport..

[30] Marcus BH, Rakowski W, Rossi JS (1992). Assessing motivational readiness and decision making for exercise. Health Psychol..

[31] Marcus BH, Rossi JS, Selby VC, Niaura RS, Abrams DB (1992). The stages and processes of exercise adoption and maintenance in a worksite sample. Health Psychol..

[32] Bradley JM, Howard JL, Wallace ES, Elborn JS (2000). Reliability, repeatability and sensitivity of the modified shuttle test in adult CF. Chest..

[33] Hu FB, Li TY, Colditz GA, Willett WC, Manson JE (2003). Television watching and other sedentary behaviours in relation to risk of obesity and type 2 diabetes mellitus in women. JAMA..

[34] Watz H, Waschki B, Boehme C, Claussen M, Meyer T, Magnussen H (2008). Extrapulmonary effects of chronic obstructive pulmonary disease on physical activity: a cross-sectional study. Am J Respir Crit Care Med..

[35] Garcia-Aymerich J, Lange P, Benet M, Schnohr P, Anto JM (2006). Regular physical activity reduces hospital admission and mortality in chronic obstructive pulmonary disease: a population based cohort study. Thorax..

[36] Gill JM, Bhopal R, Douglas A, Wallia S, Bhopal R, Sheikh A (2011). Sitting time and waist circumference are associated with glycaemia in U.K. South Asians: data from 1,228 adults screened for the PODOSA trial. Diabetes Care.

[37] Healy GN, Dunstan DW, Salmon J, Cerin E, Shaw JE, Zimmet PZ (2007). Objectively measured light-intensity physical activity is independently associated with 2-h plasma glucose. Diabetes Care..

[38] Hamilton MT, Healy GN, Dunstan DW, Zderic TW, Owen NL (2008). Too little exercise and too much sitting: inactivity physiology and the need for new recommendations on sedentary behavior. Curr Cardiovasc Risk Rep..

[39] Hagstromer M, Oja P, Sjostrom M (2007). Physical activity and inactivity in an adult population assessed by accelerometry. Med Sci Sports Exerc..

[40] Matthews CE, Chen KY, Freedson PS, Buchowski MS, Beech BM, Pate RR (2008). Amount of time spent in sedentary behaviors in the United States, 2003–2004. Am J Epidemiol..

[41] Troiano RP, Berrigan D, Dodd KW, Masse LC, Tilert T, McDowell M (2008). Physical activity in the United States measured by accelerometer. Med Sci Sports Exerc..

[42] Department of Health (2010). Health Survey for England 2008: physical activity and fitness.

[43] Hagstromer M, Troiano RP, Sjostrom M, Berrigan D (2010). Levels and patterns of objectively assessed physical activity - a comparison between Sweden and the United States. Am J Epidemiol..

[44] de Camargo AA, Amaral TS, Rached SZ, Athanazio RA, Lanza FC, Sampaio LM (2014). Incremental shuttle walking test: a reproducible and valid test to evaluate exercise tolerance in adults with noncystic fibrosis bronchiectasis. Arch Phys Med Rehabil..

[45] Newall C, Stockley RA, Hill SL (2005). Exercise training and inspiratory muscle training in patients with bronchiectasis. Thorax..

[46] Ong HK, Lee AL, Hill CJ, Holland AE, Denehy L (2011). Effects of pulmonary rehabilitation in bronchiectasis: a retrospective study. Chron Respir Dis..

[47] Mandal P, Sidhu M, Kope L, Pollock W, Stevenson LM, Pentland JL (2012). A pilot study of pulmonary rehabilitation and chest physiotherapy versus chest physiotherapy alone in bronchiectasis. Respir Med..

[48] van Zeller M, Mota PC, Amorim A, Viana P, Martins P, Gaspar L (2012). Pulmonary rehabilitation in patients with bronchiectasis: pulmonary function, arterial blood gases, and the 6-minute walk test. J Cardiopulm Rehabil Prev..

[49] Lee AL, Hill CJ, Cecins N, Jenkins S, McDonald CF, Burge AT (2014). The short and long term effects of exercise training in non-cystic fibrosis bronchiectasis - a randomised controlled trial. Respir Res..

[50] Rubak S, Sandbæk A, Lauritzen T, Christensen B (2005). Motivational interviewing: a systematic review and meta-analysis. Br J Gen Pract..

[51] Schols AMWJ, Fredrix EFHM, Soeters PB, Westerterp KR, Wouters EFM (1991). Resting energy expenditure in patients with chronic obstructive pulmonary disease. Am J Clin Nutr..

[52] Altenburg WA, Bossenbroek L, de Greef MHG, Kerjstens HAM, ten Hacken NHT, Wempe JB (2013). Functional and psychological variables both affect daily physical activity in COPD: a structural equations model. Respir Med..

