# Supplementary material for: Sedentary behaviour and physical activity in bronchiectasis: a cross-sectional study
Source: BMC Pulm Med. 2015 May 13;15:61. doi: 10.1186/s12890-015-0046-7 (PMC4456779; doi:10.1186/s12890-015-0046-7)
Supplement: Additional file 1: — Additional details of methods and materials used in the study. [file 12890_2015_46_MOESM1_ESM.docx]

**ADDITIONAL FILE 1**

**Sedentary behaviour and physical activity in bronchiectasis: a cross-sectional study**

Judy M Bradley*^1^, Jason J Wilson*^1^, Kate Hayes^1^, Lisa Kent^2^, Suzanne McDonough^1,3^, Mark A Tully^3,4^, Ian Bradbury^1^, Alison Kirk^5^, Denise Cosgrove^2^, Rory Convery^6^, Martin Kelly^7^, Joseph Stuart Elborn**^8^, Brenda O’Neill**^1^

**Joint first authors **Joint senior authors*

^1^ Centre for Health and Rehabilitation Technologies, Institute for Nursing and Health Research, Ulster University, Newtownabbey, Northern Ireland, UK

^2^ Northern Ireland Clinical Research Network: Respiratory Health, Belfast Health and Social Care Trust, Belfast, Northern Ireland, UK

^3^ UKCRC Centre of Excellence for Public Health (Northern Ireland), Belfast, Northern Ireland, UK

^4^ Centre for Public Health, School of Medicine, Dentistry and Biomedical Sciences, Queen’s University, Belfast, Northern Ireland, UK

^5^ School of Psychological Sciences and Health, University of Strathclyde, Glasgow, Scotland, UK

^6^ Southern Health and Social Care Trust, Craigavon Area Hospital, Craigavon, Northern Ireland, UK

^7^ Western Health and Social Care Trust, Altnagelvin Area Hospital, Derry, Northern Ireland, UK

^8^ Centre for Infection and Immunity, School of Medicine, Dentistry and Biomedical Sciences, Queen’s University, Belfast, Northern Ireland, UK

**Correspondence to:** Judy M Bradley, Centre for Health and Rehabilitation Technologies, Institute for Nursing and Health Research, Ulster University, Newtownabbey, Northern Ireland, UK. E-mail: [jm.bradley@ulster.ac.uk](mailto:jm.bradley@ulster.ac.uk)

**Methods**

*Participant selection*

Criteria for pulmonary exacerbation included: change in sputum production (consistency, colour, volume or haemoptysis), increased dyspnoea (chest congestion or shortness of breath), increased cough, fever (38°C), increased wheezing, decreased exercise tolerance, malaise, fatigue or lethargy, forced expiratory volume in one-second percentage predicted (FEV_1_% predicted) or forced vital capacity decreased by 10% from a previously recorded value, radiographic changes indicative of a new pulmonary process, changes in chest sounds.

A pulmonary exacerbation was defined as four or more abnormalities of the above nine symptoms + commencement of oral/IV antibiotics OR fewer than four abnormalities of the above nine symptoms + commencement of oral/IV antibiotics [1].

*Clinical measurements*

*Bronchiectasis Severity Index (BSI)*

The BSI a 9-item, clinician-completed, risk stratification tool which attempts to identify patients at risk of exacerbations, hospitalisations and mortality [2]. Using the following variables: age, BMI, FEV_1_% predicted, number of previous hospital admissions in previous 2-year period, number of exacerbations in previous year, MRC breathlessness score, pseudomonas aeruginosa colonisation (on two or more occasions at least three months apart in a 1-year period), colonisation with other organisms (on two or more occasions at least three months apart in a 1-year period) and radiological severity (number of lobes involved) – the BSI produces an overall bronchiectasis severity score. BSI scores range from 0-26. Patients can be subsequently categorised as having mild (0-4), moderate (5-8) or severe (9+) bronchiectasis. The BSI has been validated for use in bronchiectasis [2].

*Definition of ActiGraph physical activity intensities*

Time spent in sedentary behaviour and various physical activity intensities were classified using previously defined cut-off points: sedentary behaviour (<100 counts per minute / cpm), light-lifestyle physical activity (100-1951cpm) and moderate-vigorous physical activity (MVPA; ≥1952cpm) [3]. The time spent in MVPA accumulated in ≥10-minute bouts was also recorded. This variable was assessed because current physical activity guidelines acknowledge the use of bouted-MVPA measurement in the context of the individuals’ needs, goals and initial abilities; thus 30 minutes of continuous MVPA could be replaced by two or more MVPA bouts ≥10 minutes each [4]. Activity energy expenditure in kilocalories was calculated using the Freedson Combination 1998 formula (manufacturer’s algorithm). Total physical activity was assessed as daily step counts and also the sum of minutes per day in light-lifestyle physical activity and MVPA (≥100cpm) [5]. In addition, individuals’ physical activity levels were categorised using the graduated step-based physical activity index [6] This categorisation was based on research showing changes in BMI, waist-hip ratio and insulin sensitivity over five years are related to incremental changes in step-defined categories of physical activity [7]. Given the dose-response relationship between daily step counts and health outcomes, a step-based physical activity index is useful in discriminating different levels of activity below the recommended target of 10000 steps per day for the general population [8,9]. Step count data from the ActiGraph were categorised as: inactive (<5000 steps per day), low active (5000-7499 steps per day), and somewhat active and above (≥7500 steps per day).

*Quality of Life Questionnaire – Bronchiectasis (QOL-B)*

The QOL-B is a 44-item, self-completed, disease-specific HRQoL questionnaire for bronchiectasis [10]. The QOL-B has eight domains: Physical Functioning (5 items), Role Functioning (5 items), Vitality (3 items), Emotional Functioning (4 items), Social Functioning (4 items), Treatment Burden (3 items), Health Perception (4 items) and Respiratory Symptoms (9 items). Each domain was scored on a scale from 1 (worst) to 100 (best). There is no total score. The QOL-B has recently been validated with test-retest reliability found to be good [10].

*Leicester Cough Questionnaire (LCQ)*

The LCQ is a 19-item, self-completed HRQoL measure which assesses different aspects of health affected by chronic cough and is sensitive at detecting changes in health status within an adult chronic cough population [11]. The LCQ provides three domain scores: Physical (8 items), Psychological (7 items) and Social (4 items). Each domain was scored from 1 (worst) to 7 (best). These domain scores are further grouped together to produce an overall average LCQ score ranging from 3 to 21, with higher scores representing better health status. The LCQ has recently been validated for use in patients with bronchiectasis and other respiratory populations (COPD) [11].

*Stages of Change Questionnaire*

The Stages of Change Questionnaire is a 5-item, self-completed questionnaire which clarifies an individual’s current stage of change in terms of their physical activity behaviour [12]. The stages of change range from pre-contemplation (not regularly physically active and do not intend to be so in the next six months) to maintenance (regularly physically active for longer than six months). The Stages of Change Questionnaire has not been applied to bronchiectasis-specific populations. However, it has been validated with good test-retest reliability found in healthy populations [12].

*Marcus’s Self-Efficacy Questionnaire (with additional disease-specific question)*

Marcus’s Self-Efficacy Questionnaire is a 5-item, self-completed questionnaire which provides more detailed information on each individual’s confidence to undertake physical activity in different situations [13]. Items in the scale represent areas including negative affect, resisting relapse and making time for exercise. The additional disease-specific question was related to the barriers created by the typical symptoms of bronchiectasis such as chronic cough and excessive sputum production. This was added as Question Six as follows: ‘Circle the number that indicates how confident you are that you could be physically active in each of the following situations: When tired; When in a bad mood; When don’t have time; When on vacation; When raining or snowing; When having respiratory symptoms. Scores range from 1 to 5, with higher scores indicating greater confidence in being physically active in certain situations. The mean self-efficacy score was calculated from the six items. Marcus’s Self-Efficacy Questionnaire has not been applied to bronchiectasis-specific or other respiratory disease population research but has proven internal consistency and test–retest reliability within healthy populations [13].

*Marcus’s Decisional Balance Questionnaire*

Marcus’s Decisional Balance Questionnaire is a 16-item, self-completed questionnaire which assesses each individual’s perceptions of the benefits (‘pros’) and barriers (‘cons’) to physical activity [14]. Items include ‘pros’ such as having more energy and relieving tension and ‘cons’ such as having less time for family and friends and being uncomfortable when performing physical activity. Scores range from 1 to 5, with higher scores indicating greater importance. The difference between the mean ‘pros’ scores minus the mean ‘cons’ scores forms the overall decisional balance score. Positive scores indicated more perceived benefits of physical activity relative to perceived barriers while negative scores indicated more perceived barriers relative to perceived benefits. Marcus’s Decisional Balance Questionnaire has not been applied to bronchiectasis-specific or other respiratory disease population research but has been shown to have a satisfactory internal consistency for ‘pros’ and ‘cons’ in application in healthy populations [14].

*Marcus’s Processes of Change Questionnaire*

Marcus’s Processes of Change Questionnaire is a 40-item, self-completed questionnaire describing the strategies and techniques individuals use to progress through the different stages of change [15]. It comprises of 10 processes of change and these are split evenly between cognitive (increasing knowledge, being aware of risks, caring about consequences to others, comprehending benefits, increasing healthy opportunities) and behavioural strategies (substituting alternatives, enlisting social support, rewarding oneself, committing oneself and reminding oneself). Scores range from 1 to 5, with higher scores indicating greater usage of a particular strategy. Mean cognitive and behavioural processes of change scores were also calculated. This questionnaire has not been applied to bronchiectasis-specific research but has demonstrated reliability and validity in application to COPD and healthy populations [15].

**References**

1. O’Donnell C, Barker AF, Ilowite JS, Fick RB. Treatment of idiopathic bronchiectasis with aerosolized recombinant human DNase I. Chest. 1998;113:1329–34.

2. Chalmers JD, Goeminne P, Aliberti S, McDonnell MJ, Lonni S, Davidson J, et al. The Bronchiectasis Severity Index: an international derivation and validation study. Am J Respir Crit Care Med. 2014;189:576–85.

3. Freedson PS, Melanson E, Sirard J. Calibration of the Computer Science and Applications, Inc. Accelerometer. Med Sci Sports Exerc. 1998;30:777–81.

4. O’Donovan G, Blazevich AJ, Boreham C, Cooper AR, Crank H, Ekelund U, et al. The ABC of physical activity for health: a consensus statement from the British Association of Sport and Exercise Sciences. J Sports Sci. 2010;28:573–91.

5. Hart TL, Ainsworth BE, Tudor-Locke C. Objective and subjective measures of sedentary behaviour and physical activity. Med Sci Sports Exerc. 2011;43:449–56.

6. Tudor-Locke K, Bassett DR. How many steps/day are enough? Preliminary pedometer indices for public health. Sports Med. 2004;34:1–8.

7. Dwyer T, Ponsonby AL, Ukoumunne OC, Pezic A, Venn A, Dunstan D, et al. Association of change in daily step count over five years with insulin sensitivity and adiposity: population-based cohort study. BMJ. 2011;342:c7249.

8. Tudor-Locke C, Craig CL, Brown WJ, Clemes SA, De Cocker K, Giles-Corti B, et al. How many steps/day are enough for adults? Int J Behav Nutr Phys Act. 2011;8:79.

9. Ewald B, Attia J, McElduff P. How many steps are enough? Dose response curves for pedometer steps and multiple health markers in a community-based sample of older Australians. J Phys Act Health. 2013;11:509-18.

10. Quittner AL, O'Donnell AE, Salathe MA, Lewis SA, Xiaoming Li, Montgomery AB, et al. Quality of Life Questionnaire-Bronchiectasis: final psychometric analyses and determination of minimal important difference scores. Thorax. 2015;70:12–20.

11. Birring SS, Prudon B, Carr AJ, Singh SJ, Morgan MDL, Pavord ID. Development of a symptom specific health status measure for patients with chronic cough: the Leicester Cough Questionnaire (LCQ). Thorax. 2003;58:339–43.

12. Loughlan C, Mutrie N. Recruitment of sedentary NHS staff for a workplace exercise programme using an adapted “stages of change” exercise questionnaire. J Sports Sci. 1995;13:63–4.

13. Marcus BH, Selby, VC, Niaura, RS, Rossi, JS. Self-efficacy and the stages of exercise behaviour change. Res Q Exerc Sport. 1992;63:60–6.

14. Marcus BH, Rakowski W, Rossi JS. Assessing motivational readiness and decision making for exercise. Health Psychol. 1992;11:257–61.

15. Marcus BH, Rossi JS, Selby VC, Niaura RS, Abrams DB. The stages and processes of exercise adoption and maintenance in a worksite sample. Health Psychol. 1992;11:386–95.
