# Supplementary material for: Sedentary behaviour and physical activity in bronchiectasis: a cross-sectional study
Source: BMC Pulm Med. 2015 May 13;15:61. doi: 10.1186/s12890-015-0046-7 (PMC4456779; doi:10.1186/s12890-015-0046-7)
Supplement: Additional file 2: — Comparing sedentary behaviour and physical activity in patients with bronchiectasis to healthy and COPD populations. [file 12890_2015_46_MOESM2_ESM.docx]

**ADDITIONAL FILE 2**

**Sedentary behaviour and physical activity in bronchiectasis: a cross-sectional study**

Judy M Bradley*^1^, Jason J Wilson*^1^, Kate Hayes^1^, Lisa Kent^2^, Suzanne McDonough^1,3^, Mark A Tully^3,4^, Ian Bradbury^1^, Alison Kirk^5^, Denise Cosgrove^2^, Rory Convery^6^, Martin Kelly^7^, Joseph Stuart Elborn**^8^, Brenda O’Neill**^1^

**Joint first authors **Joint senior authors*

^1^ Centre for Health and Rehabilitation Technologies, Institute for Nursing and Health Research, Ulster University, Newtownabbey, Northern Ireland, UK

^2^ Northern Ireland Clinical Research Network: Respiratory Health, Belfast Health and Social Care Trust, Belfast, Northern Ireland, UK

^3^ UKCRC Centre of Excellence for Public Health (Northern Ireland), Belfast, Northern Ireland, UK

^4^ Centre for Public Health, School of Medicine, Dentistry and Biomedical Sciences, Queen’s University, Belfast, Northern Ireland, UK

^5^ School of Psychological Sciences and Health, University of Strathclyde, Glasgow, Scotland, UK

^6^ Southern Health and Social Care Trust, Craigavon Area Hospital, Craigavon, Northern Ireland, UK

^7^ Western Health and Social Care Trust, Altnagelvin Area Hospital, Derry, Northern Ireland, UK

^8^ Centre for Infection and Immunity, School of Medicine, Dentistry and Biomedical Sciences, Queen’s University, Belfast, Northern Ireland, UK

**Correspondence to:** Judy M Bradley, Centre for Health and Rehabilitation Technologies, Institute for Nursing and Health Research, Ulster University, Newtownabbey, Northern Ireland, UK. E-mail: [jm.bradley@ulster.ac.uk](mailto:jm.bradley@ulster.ac.uk)

**Table S1:** Comparing sedentary behaviour and physical activity in patients with bronchiectasis to healthy and COPD populations

| **Variables** | **Bronchiect-asis data (wear-time: 865 mins)** | **England healthy [1]**  **(M wear-time: 849 mins;**  **F wear-time: 830 mins)** | **Sweden** **[2,3] healthy (wear- time: 830 mins)** | **USAA760994886 judyons: nisms (on two NHANES healthy**  **[3,4,5,6]**  **(wear-time: N/A)** | **USA NHANES COPD [7]**  **(wear-time: 932 mins)** |
| --- | --- | --- | --- | --- | --- |
| Sedentary  behaviour (mins/day) | 634 (77)  73% | M:595 (83)*  70%  F:584 (82)*  70% | 459 (86)*  55% | 460 (191)* | 676 (170)  72% |
| Light- lifestyle physical activity (mins/day) | 207 (63)  24% | M:212 (73)  25%  F:231 (74)*  28% | M:320 (N/A)  39%^a^ F:334 (N/A)^a^  40% | M:293 (N/A)^a^ F:321 (N/A)^a^ | 250 (98)*  27% |
| Total MVPA (mins/day) | 25 (20)  3% | M:31 (30)  4%  F:24 (22)  3% | 31 (33)  4% | M:17 (20)*^b^ F:12 (15)*^b^ | 6 (11)*  1% |
| MVPA_10+_ (mins/day) | 6 (9) | M:11 (18)*  F:8 (17) | M:16 (10)*^a^ F:13 (11)*^a^ | M:7 (18)^b^ F:6 (15)^b^ | N/A |
| Daily step counts | 6001 (2780) | N/A | N/A | 9676 (6547)* | N/A |

Results are Mean (SD).

Where wear-time data was available, % time spent in different physical activity intensities during waking hours was calculated.

Abbreviations: F - female; M - male; mins - minutes; mins/day - minutes per day; MVPA - moderate-vigorous physical activity; MVPA_10+_ - MVPA accumulated in ≥10-minute bouts; N/A - Not available; NHANES - National Health and Nutrition Evaluation Survey; US - United States.

* significant difference to bronchiectasis data (p<0.05).

^a^ No all-age data collated (60 - 75 year olds chosen); ^b^ No all-age data collated (60 - 69 year olds chosen).

**References**

1. Department of Health: Health Survey for England 2008: physical activity and fitness. London: The Stationery Office; 2010.

2. Hagstromer M, Oja P, Sjostrom M. Physical activity and inactivity in an adult population assessed by accelerometry. Med Sci Sports Exerc. 2007;39:1502–8.

3. Hagstromer M, Troiano RP, Sjostrom M, Berrigan D. Levels and patterns of objectively assessed physical activity - a comparison between Sweden and the United States. Am J Epidemiol. 2010;171:1055–64.

4. Matthews CE, Chen KY, Freedson PS, Buchowski MS, Beech BM, Pate RR, Troiano RP. Amount of time spent in sedentary behaviors in the United States, 2003–2004. Am J Epidemiol. 2008;167:875–81.

5. Tudor-Locke C, Johnson WD, Katzmarzyk PT. Accelerometer-determined steps per day in US adults. Med Sci Sports Exerc. 2009;41:1384–91.

6. Troiano RP, Berrigan D, Dodd KW, Masse LC, Tilert T, McDowell M. Physical activity in the United States measured by accelerometer. Med Sci Sports Exerc. 2008;40:181–8.

7. Park SK, Richardson CR, Holleman RG, Larson JL. Physical activity in people with COPD, using the National Health and Nutrition Evaluation Survey (NHANES) dataset (2003–2006). Heart Lung. 2013;42:235–40.
